# Can your house keep you out of a nursing home?

**DOI:** 10.1002/hec.4001

**Published:** 2020-01-31

**Authors:** Maaike Diepstraten, Rudy Douven, Bram Wouterse

**Affiliations:** ^1^ CPB The Netherlands Bureau for Economic Policy Analysis The Hague The Netherlands; ^2^ Erasmus School of Health Policy & Management Erasmus University Rotterdam Rotterdam The Netherlands

**Keywords:** accessibility of houses, ageing, demand for long‐term care, elderly housing, nursing home care

## Abstract

We examine the impact of the accessibility of an older individual's house on her use of nursing home care. We link administrative data on the accessibility of all houses in the Netherlands to data on long‐term care use of all older persons from 2011 to 2014. We find that older people living in more accessible houses are less likely to use nursing home care. The effects increase with age and are largest for individuals aged 90 or older. The effects are stronger for people with physical limitations than for persons with cognitive problems. We also provide suggestive evidence that older people living in more accessible houses substitute nursing home care by home care.

## 1 INTRODUCTION

The housing environment is vital to the quality of life of older individuals (Harvard Joint Center for Housing Studies, 2014). Across the developed world, there is a tendency for older people to keep living in their own home, even at high ages and with severe limitations (Nederlandse Zorgautoriteit, 2018; OECD, 2017). This trend in “ageing in place” seems to be driven by the preferences of older individuals themselves, who would like to keep living in their own home as long as possible (e.g., Binette & Vasold, 2018; Costa‐Font, Elvira, & Mascarilla‐Miró, 2009; World Health Organization, 2011), and by a desire of policy makers to save expenditures by substituting nursing home care by hopefully less costly home care (OECD, 2017). 1The evidence whether substitution from nursing homes to home care is indeed cost saving is mixed and seems to be context depended (Bakx et al., 2018; Blackburn, Locher, & Kilgore, 2014; Kok et al., 2015; Naomi, Shiroiwa, Fukuda, & Murashima, 2012; Young et al., 2017).


Policy makers actively try to stimulate ageing in place through improvements in the house. Governments often provide subsidies within their (social) long‐term care insurance system for home improvements. In the Netherlands, for instance, older people can directly receive home modifications, such as a stair lift, financed by the municipality.

Although housing improvements are thus perceived as contributing to ageing in place, it is the question whether the quality of the house really is an important factor for living longer at home and postponing nursing home care. There is a vast literature on the determinants of nursing home care such as age, morbidity, partner status, and income (De Meijer, Koopmanschap, Bago d'Uva, & Van Doorslaer, 2011; Kim, Kwon, Yoon, & Hyun, 2013; Luppa, Luck, Matschinger, König, & Riedel‐Heller, 2010; Luppa, Luck, Weyerer, et al., 2010; Portrait, Lindeboom, & Deeg, 2000; Rapp et al., 2015; Rouwendal & Thomese, 2013; Slobbe, Wong, Verheij, van Oers, & Polder, 2017; Wong, Elderkamp‐de Groot, Polder, & van Exel, 2010). However, large scale evidence on the role of the accessibility of the house on the use of nursing home care is scarce. 2Bockarjova et al. (2016) examine the link between the house and nursing home care on a smaller set of observations by using survey data. The aim of this study is to fill this gap and to provide empirical evidence on whether the accessibility of the house postpones, or prevents, older persons from using nursing home care.

There are two channels through which the accessibility of the house can affect the use of nursing home care. First, it can have a direct effect on the health of older individuals. Housing characteristics, such as uneven floors, absence of hand rails, and inconvenient doorsteps, have been found to be related to falls (Aarsland, Larsen, Tandberg, & Laake, 2000; Isberner et al., 1998; Larsen, Mosekilde, & Foldspang, 2004). In turn, falls are an important reason for nursing home admission (American Geriatrics Society et al., 2001; Rubenstein, 2006; Tinetti & Williams, 1997; Wolinsky, Johnson, & Fitzgerald, 1992).

Second, older people who live in more accessible houses might be better able to cope with their limitations. Particular functional limitations might be less important for the daily functioning of someone living in an accessible than inaccessible house. Not being able to walk up the stairs, for instance, is less of a problem for someone who lives on the ground floor. Thus, the physical environment of the home has a prosthetic function (Sanford, 2010). There is evidence that older people with access to assistance devices in their home are better able to cope with limitations and need less hours of help (Agree, Freedman, Cornman, Wolf, & Marcotte, 2005; Freedman, Agree, Martin, & Cornman, 2006; Hoenig, Taylor, & Sloan, 2003). The physical environment of the house can also have a therapeutic function by supporting the provision of health care services (Sanford, 2010). As explained by Aedes‐Arcares (2005), home care might be more easily provided in a house that is accessible, which might reduce the need for nursing home care. Bockarjova, Polder, and Rouwendal (2016) show that living in an elderly dwelling is indeed associated with more long‐term care consumption at home. Furthermore, unsafe environments can negatively impact care providers, leading to less provision of care (Sanford, 2010).

In this study, we investigate whether older people living in more accessible, or easily adaptable houses, are less likely to use nursing home care during the year than older people in less accessible houses. We do this by using data on the accessibility of almost all houses in the Netherlands, based on a star rating. We link these data to administrative data on individual nursing home admissions, income and wealth, and health care use of all older people in the Netherlands. We confirm the plausibility for our main estimates by performing three additional analyses. We differentiate between nursing home care use based on physical and cognitive grounds, we assess the effects of accessibility on the use of home care, and we perform an instrumental variable analysis based on the house the individual lived in 15 years ago.

## 2 THE DUTCH LONG‐TERM CARE SYSTEM

The Netherlands has one of the most extensive public long‐term care systems in the world (OECD, 2017). In our study period (before 2015), all inhabitants of the Netherlands were insured under a social insurance called the Exceptional Medical Expenses Act (AWBZ). This insurance covered all chronic care and included a broad range of home care services and institutional care for older individuals. 3Daily housekeeping activities were not part of the AWBZ, as it was shifted to the Law on Social Assistance (WMO) in 2007. Users had to pay a copayment for both home care and nursing home care. This copayment depended on the type of care and the amount of care used. The monthly copayment was maximized, depending on income and financial wealth, guaranteeing that long‐term care was accessible for all income groups. 4For nursing home care, the system distinguishes between low copayments for persons with expenditures outside the institution (for example, when the stay in the institution is temporary, or when a partner still lives at home) and high copayments for others. In 2014, the low copayment ranged between 156 and 819.40 euro a month, and the high copayment was maximized at 2,248.60 per month. We do not directly measure copayments, but we control for income and financial wealth in all analyses.


Institutional care was provided in a residential or nursing home setting (Bakx, Wouterse, van Doorslaer, & Wong, 2018; Kok, Berden, & Sadiraj, 2015). The setting and intensity differed depending on the needs and health problems. Throughout the paper, we will refer to both as nursing home care. Home care is formal care provided at home. This included domiciliary care (assistance with dusting, vacuuming, and cleaning windows), personal care (assistance with washing, dressing, and eating), nursing (providing care at home), and personal assistance (help with financial affairs and social contacts). Persons could also receive group assistance outside their own home (adult day care, such as help with day structuring or activities). 5Eligibility to specific types of home care is granted based on the need of the patients, and different types of care can be provided by different professionals.


Eligibility for nursing home care was based on a need for care due to health problems and disability. The grounds for eligibility could be either physical (somatic, physical, or sensory) or nonphysical (psychogeriatric, psychiatric, or mental). Although the grounds for care were thus always health related, the assessor was required to take the social environment and living conditions (including the house) into account when determining the type and amount of care an individual was entitled to.

## 3 METHODS

### 3.1 The basic model

We model the effect of the accessibility of the house on the use of nursing home care. In our main analysis, we are interested in whether an individual *i* uses any nursing home care in year *t*, *y*_*i*,*t*_ = {0,1}. We think of the use of nursing home care in terms of an individual's latent health needs $y_{i,t}^{*}$. An individual will use nursing home care if her latent health needs are higher than some threshold: $y_{i,t}^{*}>\alpha$. The threshold depends both on an individual's own preferences (to request eligibility) and on the eligibility criteria used by the assessment agency.

We model latent health needs of an individual as a linear function of observed individual characteristics (*X*_*i*,*t*_)*,* the accessibility of the house, and an unobserved component:

$y_{i,t}^{*}$= $X_{i,t}^{'}\beta +\gamma s_{i,t}+\varepsilon_{i,t}$.

For now, it is easiest to think of the accessibility of the house *s*_*i*,*t*_ as a dummy variable, which is 1 if the individual's house is well accessible, and 0 if a house is poorly accessible. The parameter of interest is *γ*. The hypothesis that we will test throughout this paper is whether *γ*<0; the propensity to use nursing home care is lower for people in well accessible houses. The effect we estimate is a combined effect of the direct effect of the house on health and the effect of the house on the ability to cope with limitations.

In terms of observed nursing home care use *y*_*i*,*t*_, we can write the model as

(1) $$\begin{array}{l} y_{i,t}^{*}=X_{i,t}^{'}\beta +\gamma s_{i,t}+\varepsilon_{i,t}\leq \alpha \Rightarrow y_{i,t}=0\\ y_{i,t}^{*}=X_{i,t}^{'}\beta +\gamma s_{i,t}+\varepsilon_{i,t}>\alpha \Rightarrow y_{i,t}=1. \end{array}$$

We assume that the unobserved part *ε*_*i*,*t*_~*N*(0, *σ*), so that we can estimate the following linear probability model:

(2) $$y_{i,t}=\alpha +X_{i,t}^{'}\beta +\gamma s_{i,t}+\varepsilon_{i,t}.$$

We estimate the use of nursing home care in a particular year *t* as a function of an individual's characteristics, neighborhood and housing characteristics, and the accessibility of her house at the start of year *t.* We have an unbalanced panel. Most individuals appear every year in our dataset, that is, individuals that do not use nursing home care and do not die. We exclude individuals who already live in a nursing home at the start of the year. 6For persons living in a nursing home at the start of year *t*, the accessibility of the home would measure the accessibility of the nursing home. We cluster the standard errors at the individual level.

In the estimation, we make three changes to the simplified model in Equation (1). First, we distinguish four types of houses. This means that we have to include three dummies and estimate three effects (relative to the reference category). We expect the strongest (most negative) effect for most accessible houses. Also, in addition to Equation (1), we relax the assumption of a constant effect, by letting *s*_*i*,*t*_ interact with age. 7We use age as a proxy for health characteristics of older people that are not observable in our administrative data. For example, also healthy older people with no medical history will become frailer at older ages. As most health problems correlate with age (De Meijer, Wouterse, Polder, & Koopmanschap, 2013), we expect the effect of the house on nursing home care use to increase with age as well.

Second, we control for a wide range of characteristics by including in *X*_*i*,*t*_ dummies for age, gender, type of home care used in the previous year, different types of medicines, the logarithms of various healthcare expenditures, being a home owner, having a partner, having children, having children living at home, gross income deciles, and financial wealth 8The financial wealth variable captures capital and liabilities that are not related to the house. deciles. Moreover, we control for country of birth and include the distance an individual lives to several services in the living environment, such as a supermarket or a general practitioner.

Third, to control for the fact that older people with poor (unobserved) health might be clustered in particular neighborhoods, we include neighborhood fixed effects (*δ*_*j*_) in the regression model:

(3) $$y_{i,t}=\alpha +\delta_{j}+X_{i,t}^{'}\beta +\gamma s_{i,t}+\varepsilon_{i,t}.$$

Subscript *j* denotes the neighborhood individual *i* lives in. We distinguish approximately 11,000 neighborhoods. The average number of older persons living in a neighborhood equals 79. We now identify the effect of *s*_*i*,*t*_ solely on the variation in the accessibility of the houses within neighborhoods.

### 3.2 Additional analyses

To test the plausibility of our estimates and obtain more insight in the mechanisms that drive the effect of the accessibility of the house on nursing home care use, we extend our analysis in three ways.

First, we investigate whether the effect of the accessibility of the house is related to the reason why people are eligible for nursing home care. Eligibility can be granted either on physical (somatic, physical, or sensory) or cognitive (psychogeriatric, psychiatric, or mental) ground. Although persons can suffer from both physical and cognitive problems (Tolea, Moris, & Galvin, 2016), we hypothesize that the type of house matters more for persons whom the primary ground is physical than for persons whom the primary ground is cognitive. In order to investigate this hypothesis, we estimate Equation (3) twice. Ones for *y*_*i*,*t,phy*_, a dummy that is equal to 1 when individual *i* uses nursing home care in *t* on physical grounds, and 0 when the individual does not use nursing home care. And ones for *y*_*i*,*t,cog*_, that is 1 if an individual uses nursing home care in year *t* on cognitive grounds, and 0 if one does not use nursing home care.

The second mechanism we explore is whether older people living in accessible houses use less nursing home care because they have more possibilities to use home care instead. Thus, does the accessibility of the house stimulate substitution from nursing home care to home care? We estimate Equation (3), but now with the use of home care (yes/no) as the dependent variable. We exclude persons who used homecare in the prior year to focus on new cases.

Third, older people might selectively move to an accessible house because they have health problems. As a result, older people who live in an accessible house might have poorer unobserved health than older persons living in a poorly accessible house. This would bias our estimate of *γ* upwards. To investigate whether selective moving is at play, we perform an instrumental variable analysis. We use the *current* accessibility of the house, an individual lived in 15 years ago, as an instrument for the accessibility of the house an individual currently lives in. 9Because of data availability, we focus on the house 15 years ago.
^,^ 10We have three endogenous variables, and we also have three instruments: we create dummies for each type of house, which is equal to 1 if someone lived in such type of house 15 years ago. The identifying assumption is that, in making their housing decision at *t‐*15, individuals did not have or use information on their long‐term care needs at *t.* The two‐stage model then becomes

(4) $$s_{i,t}=\kappa +\delta_{j}+X_{i,t}^{'}\theta +\lambda s_{i,t-15}+\nu_{i,t},$$

(5) $$y_{i,t}=\alpha +\delta_{j}+X_{i,t}^{'}\beta +\gamma {\hat{s}}_{i,t}+\varepsilon_{i,t},$$

where ${\hat{s}}_{i,t}$ in the second stage Equation (5) is the predicted probability of living in an accessible house at time *t,* based on the first stage Equation (4). We have four different types of houses and four age categories. This means that there are 12 endogenous variables. Hence, we run 12 first stage regressions on the instruments, the interactions between the instruments and the age dummies, and all control variables. We combine the fixed effects and IV model using the estimator of Correia (2016), which is developed to estimate large linear probability models with many fixed effects.

## 4 DATA

### 4.1 Data sources

We use a novel dataset on the accessibility of almost all buildings in the Netherlands (TNO). 11TNO, the Netherlands Organization for applied scientific research, is an independent research organization. Information about the accessibility of individual buildings is publicly available. For details about the dataset, see http://www.zorgopdekaart.nl/bagwoningen/pdfs/toelichting/toelichting-woningvoorraad-woningaanpassingen-en-langer-zelfstandig-thuis-11.pdf
 The dataset is created in 2016 and shows to what extent a house is accessible, or can be made accessible by acceptable costs, for people with mobility problems.

**Figure 1:**
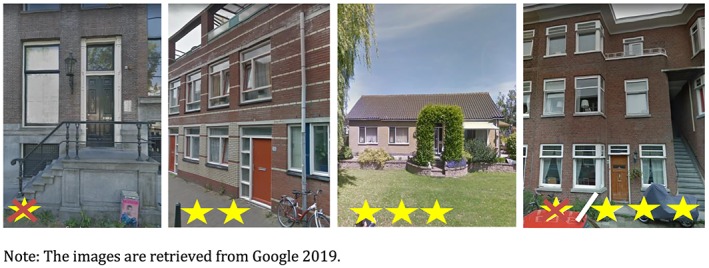
Examples of respectively a 0‐star, 2‐star, 3‐star, and 0/3‐mix house [Colour figure can be viewed at http://wileyonlinelibrary.com]

All houses are classified with 0, 2, or 3 stars or 0/3‐mix (Figure 1). A 0‐star house is a poorly accessible house: one has to climb stairs to reach the front door, and hence, it cannot be made accessible by acceptable costs. A 2‐star house can be accessed without taking the stairs, consists of multiple floors, and it is possible to place a stair lift at acceptable costs (less than 10,000 euros). A 3‐star house is the most accessible house: it can be accessed without walking the stairs, and it has only one floor. For apartments in a building without an elevator, it is not always known whether the apartment is on the ground floor (3‐star house) or above (0‐star house). Therefore, all apartments in such building are placed in the category 0/3‐mix. 12In theory, there are also 1‐, 4‐, and 5‐star houses; 1‐star houses are houses of which the front door is accessible without stairs, which has multiple levels, and where it is not possible to place a stair lift. In practice, it is unknown whether the stairwell is large enough for a stair lift, but as this is the case for 95% of houses, it is assumed that it is always possible. Consequently, we do not observe 1‐star houses in the data; 4‐ and 5‐star houses are connected to a care institution but are not observed in the data.


We link the dataset to individual‐level administrative data for the years 2011–2014 from different sources. We have data on all positive eligibility decisions for nursing home care and home care data obtained from the Dutch assessment agency (CIZ), including the grounds for the assessment (physical or nonphysical). We also have data on all care episodes for use of home care and nursing home care, including the date of admission (data obtained from the Central Administration Office, CAK). Furthermore, we link the data to data on prior health care use (type of home care in the previous year, medicine use, 13Data from Health Care Insurance Board (College voor Zorgverzekeringen in Dutch). and costs of curative care used under the mandatory basic health care insurance 14Data from Vektis.), personal characteristics (age, gender, country of birth, partner status, household type, number of children, current and living address 15 years ago 15Data from the Dutch population register., neighborhood 16Data from TNO., income, net financial and housing wealth, and house ownership 17Data from the tax authority.), and facilities in the surroundings 18Data from Statistics Netherlands. (distance of house to nearest supermarket, general practice, general practice center, pharmacy, and hospital).

### 4.2 Sample selection

We have data on 2011–2014. For all the time varying explanatory variables, we use lagged values in our regression. That is, we include medicine use, use of home care, curative care expenditures, and gross income in year *t‐*1. We include whether one has a partner and has children living at home at December 31 of year *t‐*1, and we include age, financial wealth, the accessibility of the house, and home ownership at January 1 of year *t.*

We restrict the sample to persons of 75 years and older. This leaves us with 4,187,202 observations. We exclude patients with a care package for restorative care, since 2013, a part of these patients received care out of the curative care insurance (Health Insurance Act). Besides, we exclude patients with a care package for palliative care. We retain 3,861,608 observations. Furthermore, we exclude individuals who used nursing home care in the past year leaving us with 3,465,436 observations. After excluding observations for which the accessibility of the home is unknown, we retain 2,793,545 observations. Because of missing values in other variables, we end with a dataset comprising of 2,599,069 observations to estimate nursing home care use for 2012–2014.

### 4.3 Descriptive statistics

**Figure 2:**
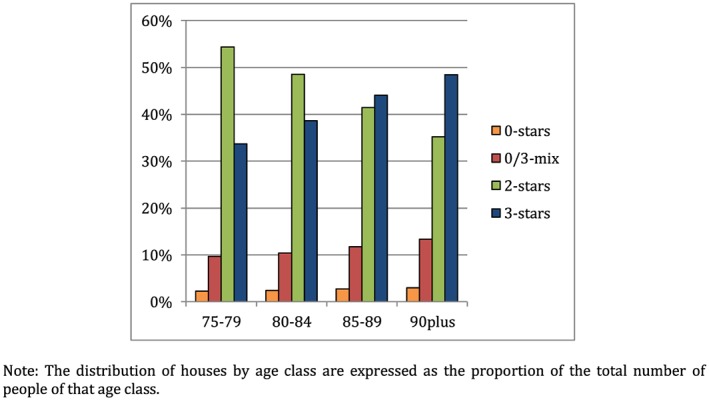
Distribution of houses by age class [Colour figure can be viewed at http://wileyonlinelibrary.com]

Table 1 displays descriptive statistics of the observations included in the main regression specification (Column 1 in Table 2) by type of house for 2012–2014. Two percent live in a 0‐star house, 11% live in a 0/3‐mix house, 49% live in a 2‐star house, and 38% live in a 3‐star house. Figure 2 shows that the majority of the persons in the oldest age groups live in a 3‐star house, whereas the majority of the younger age groups live in a 2‐star house. The proportion of people living in a 0‐star house is fairly constant across age groups.

**Table 1:** Descriptive statistics by type of house

	0‐star house		0/3‐mix house		2‐star house		3‐star house	
	Mean	SD	Mean	SD	Mean	SD	Mean	SD
Total number of observations	64,171		274,752		1,262,400		997,746	
Use of nursing home care	0.04		0.03		0.02		0.03	
Assessment on physical ground	0.02		0.02		0.01		0.02	
Assessment on cognitive ground	0.01		0.01		0.01		0.01	
Use of home care	0.35		0.40		0.29		0.40	
Age 75–79	0.37		0.36		0.44		0.35	
Age 80–84	0.35		0.35		0.35		0.35	
Age 85–89	0.20		0.20		0.15		0.21	
Age 90plus	0.09		0.09		0.05		0.09	
Male	0.37		0.37		0.46		0.39	
Dutch	0.86		0.89		0.94		0.94	
Having a partner	0.36		0.36		0.53		0.43	
Having children	0.80		0.84		0.90		0.86	
Having children living at home	0.05		0.04		0.07		0.03	
Gross income	28,712	17,130	30096	24,100	37303	29,746	32971	24,191
Financial wealth	71,394	253,187	101357	456,071	130430	596,660	112141	474,617
Medicine user	0.95		0.96		0.95		0.96	
Cholestorol reducer	0.39		0.40		0.40		0.40	
Diabetes	0.18		0.19		0.16		0.18	
Astma	0.17		0.19		0.17		0.18	
Antidepressants	0.09		0.10		0.08		0.10	
Antipsychotics	0.03		0.03		0.03		0.03	
Sleeping and tranquilizing tablets	0.07		0.07		0.05		0.06	
ADHD and nootropics	0.00		0.00		0.00		0.00	
Other medicines	0.94		0.95		0.94		0.96	
Log of total ZVW expenditures (without mental health care costs) in euros	7.58	1.34	7.67	1.33	7.59	1.32	7.71	1.28
Log of total GP care costs in euros	5.36	0.56	5.42	0.59	5.37	0.56	5.43	0.57
Log of total pharmaceutical care costs in euros	5.67	1.78	5.80	1.72	5.63	1.76	5.85	1.65
Log of total oral care costs in euros	0.48	1.63	0.49	1.64	0.49	1.64	0.51	1.67
Log of total hospital care costs in euros	5.89	2.79	6.03	2.70	5.97	2.69	6.13	2.60
Log of total paramedical care costs in euros	0.44	1.59	0.52	1.73	0.47	1.66	0.54	1.76
Log of total technical aids costs in euros	2.60	2.98	2.86	3.04	2.48	2.99	2.92	3.06
Log of total patient transport costs in euros	0.87	2.23	0.91	2.29	0.75	2.12	0.89	2.26
Log of total health care costs outside the Netherlands in euros	0.11	0.80	0.11	0.79	0.09	0.71	0.08	0.69
Log of total other care costs in euros	1.75	2.40	1.91	2.46	1.84	2.49	1.92	2.48
Domiciliary care in prior year	0.23		0.26		0.16		0.26	
Personal care in prior year	0.14		0.19		0.13		0.20	
Nursing in prior year	0.06		0.07		0.05		0.08	
Personal assistance in prior year	0.02		0.02		0.01		0.02	
Group assistance in prior year	0.02		0.03		0.02		0.03	
Home owner	0.20		0.25		0.58		0.35	
Living within 500 m of supermarket	0.59		0.57		0.37		0.44	
Living within 500 m of general practice	0.49		0.50		0.32		0.38	
Living within 500 m of general practice center	0.01		0.01		0.01		0.01	
Living within 500 m of pharmacy	0.44		0.42		0.23		0.30	
Living within median distance to hospital	0.77		0.63		0.44		0.55	

*Note.* Descriptive statistics are based on the observations included in the main regression analysis (Column 1 in Table 2) measured at time *t* for the years 2012–2014. The standard deviation (SD) is reported for continuous variables only.

**Table 2:** Use of nursing home care

	1	2	3
	Use of nursing home care	Use of nursing home care	Use of nursing home care
0/3 mix	0.054	−0.188**	0.292*
	(0.085)	(0.078)	(0.149)
2 stars	0.126	−0.177**	0.399***
	(0.079)	(0.072)	(0.136)
3 stars	−0.009	−0.280***	0.389***
	(0.080)	(0.072)	(0.138)
0/3 mix * Age 80–84	−0.253	−0.259*	−0.407
	(0.156)	(0.155)	(0.261)
0/3 mix * Age 85–89	−0.777***	−0.773***	−0.717*
	(0.262)	(0.262)	(0.409)
0/3 mix * Age 90 plus	−0.972*	−0.933*	−1.803**
	(0.521)	(0.520)	(0.780)
2 stars * Age 80–84	−0.474***	−0.461***	−0.766***
	(0.143)	(0.142)	(0.233)
2 stars * Age 85–89	−1.242***	−1.251***	−1.494***
	(0.243)	(0.243)	(0.368)
2 stars * Age 90 plus	−2.230***	−2.169***	−3.680***
	(0.488)	(0.488)	(0.719)
3 stars * Age 80–84	−0.352**	−0.351**	−0.515**
	(0.145)	(0.144)	(0.242)
3 stars * Age 85–89	−0.992***	−1.038***	−0.770**
	(0.244)	(0.244)	(0.382)
3 stars * Age 90 plus	−1.596***	−1.649***	−2.438***
	(0.485)	(0.485)	(0.737)
Observations	2,599,069	2,599,069	2,466,684
*R*‐squared	.079	.073	.073
Year dummies	YES	YES	YES
Neighborhood fixed effects	YES	NO	YES
Specification	OLS	OLS	IV

*Note.* The regression results explain the use of nursing home care. The coefficients are multiplied by 100 and hence express percentage points. All specifications include health controls, personal characteristics, and neighborhood characteristics.

Abbreviation: OLS, ordinary least squares.

*** *p* < .01.

** *p* < .05.

* *p* < .1.

Table 1 shows that the fraction of people who go to nursing home in the following year is relatively small. This is 4% for people living in a 0‐star house, 3% for a 0/3‐mix house, 2% for a 2‐star house, and 3% for a 3‐star house. One to two percent of the people receive an assessment on physical ground and 1% an assessment on cognitive ground, irrespective of the type of house.

Other interesting results are that persons living in the most accessible houses are more likely to have a partner than older people living a 0/3‐mix or 0‐star house. People living in a 2‐star house are mostly likely to own the house and have a higher gross income and more financial wealth than others. Almost all persons use some kind of medicines, irrespective of the type of house. Persons living a 2‐star house are less likely to have used home care in the past year. People living in the least accessible houses are most likely to live close to a supermarket, general practitioner, pharmacy, and hospital than others. Only 1% of persons live within 500 m of a practitioner center.

## 5 RESULTS

### 5.1 Use of nursing home care

Table 2 shows the main regression coefficients to explain variation in the use of nursing home care. Appendix A contains the complete regression output including the coefficients of all control variables. The first column of Table 2 presents the result of an ordinary least squares regression with neighborhood fixed effects. This is our preferred specification.

**Figure 3:**
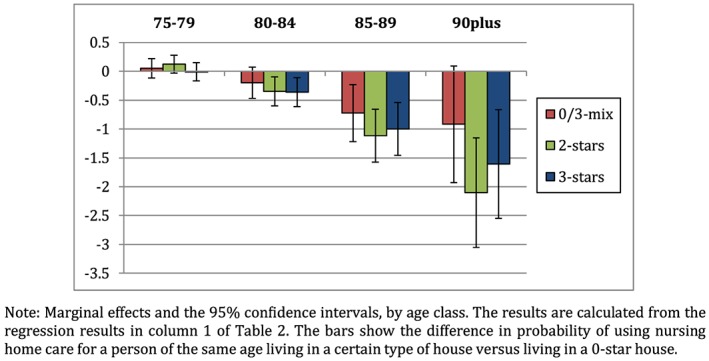
The marginal effects of the accessibility of the house on the use of nursing home care [Colour figure can be viewed at http://wileyonlinelibrary.com]

To examine whether the house plays a role in using nursing home care, we calculate the marginal effects, shown in Figure 3. 19A marginal effect is calculated as the sum of the coefficient of the house and the coefficient of the interaction term of the house and age. We use the non‐rounded coefficients to construct the figure and hence the values may slightly deviate from the sum of the coefficients shown in Table 2. The figure shows the difference in the probability to use nursing home care between a person living in a certain type of house versus (2 star, 3 star, or 0/3‐mix) a person with similar characteristics and the same age living in a 0‐star house (the reference category).

The main results are in line with our hypotheses. First, people living in well accessible houses use less nursing home care than people living in poorly accessible houses. This is the case across all ages. The effects of houses that can be made accessible (a 2‐star house) are similar in size to houses that are accessible (a 3‐star house). The effect of a 0/3‐mix house lies between the effects of a 0‐star house and a 3‐star house, which is intuitive as this category comprises of 0‐star houses and 3‐star houses.

Second, the effect of the house increases with age and is strongest for the oldest age groups. For example, consider persons living in a 2‐star house. For people between 75 and 79 years old, the probability to nursing home care is not statistically different at 5% from individuals of that age living in a 0‐star house. In contrast, persons between 80 and 84 year old are 0.3 percentage points less likely to use nursing home care than persons of the same age living in a 0‐star house, and persons of 85–89 years old and persons aged 90 plus are, respectively, 1.1 and 2.1 percentage points less likely to use nursing home care (all significant at 5%). The results become more significant at higher ages: although the interaction coefficients and the marginal effects are not statistically different from 0 for persons between 75 and 79 years old, most of the results are significant for persons aged 80 and over.

Appendix A outlines the complete regression results and shows that the effects of the control variables are in line with the literature: Older people and people with worse health status are more likely to use nursing home care, and individuals who own their house, who have a higher gross income, and who have children are less likely to use nursing home care. Excluding neighborhood fixed effects hardly affects the estimates (Column 2 in Table 2): the coefficients of the interaction terms are comparable in size and significance levels.

### 5.2 Additional analyses

We perform three additional analyses to test the plausibility of our estimated effects. In this section, we discuss the most relevant outcomes. We refer to Appendix C for the regression coefficients. The complete estimation results are available on request.

**Figure 4:**
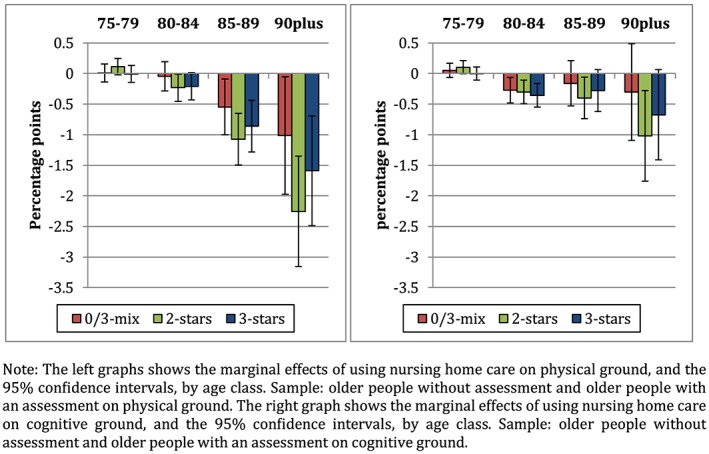
Marginal effects on the use of nursing home care on physical grounds (left) and the marginal effects on the use of nursing home care on cognitive grounds (right) [Colour figure can be viewed at http://wileyonlinelibrary.com]

We first investigate whether the effect of the accessibility of the house is larger for individuals with physical health problems than with cognitive health problems. The left graph in Figure 4 presents the marginal effects of using nursing home care on a physical ground and the right graph in Figure 4 of using nursing home care on a cognitive ground. As in the main analysis, in both cases, the effect of the accessibility of the house increases with age. In line with our hypothesis, the effects are (more than twice) larger for persons with physical problems as for people with cognitive limitations for individuals of 85 years and older.

**Figure 5:**
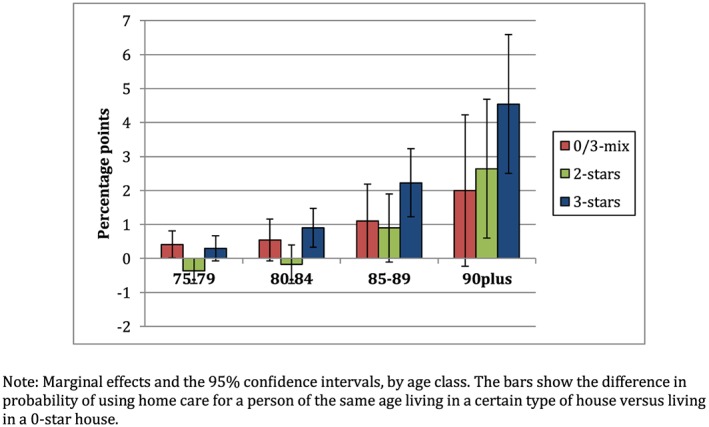
Marginal effects on the use of home care [Colour figure can be viewed at http://wileyonlinelibrary.com]

Second, we examine whether living in an accessible house leads to substitution of nursing home care by home care. We run similar regressions as before, but now with home care use as dependent variable. Figure 5 shows the marginal effects. As we hypothesized, persons living in more accessible houses are more likely to use home care. We find significant positive effects on the probability of home care use for 3‐star houses in the age group 85–89 and 2‐star and 3‐star houses in the ages above 90. For the 3‐star houses, the positive effect on home care use (in percentage points) is larger than the negative effect on nursing home care. A possible explanation for this difference is that living in an accessible house might also enable individuals, who would otherwise not receive any formal care, to receive care at home (Aedes‐Arcares, 2005). 20This mechanism might also be related to selective moving: individuals might move to an accessible house in order to be able to receive home care. Indeed, when we try to control for selective moving using an IV analysis (available upon request), we do not a significant difference in total use of care (home care or nursing home care) between the star ratings.


**Figure 6:**
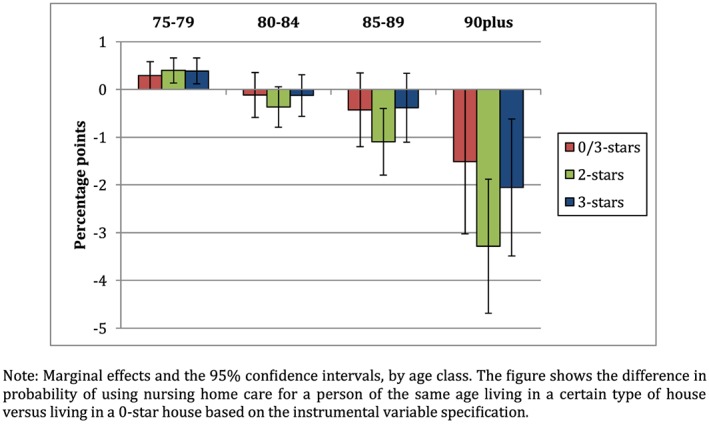
Marginal effects on the use of nursing home care (IV) [Colour figure can be viewed at http://wileyonlinelibrary.com]

Third, we perform an instrumental variable analysis, to test whether selective moving of older individuals with health problems to accessible houses affects our results. We use the *current* accessibility of the house, an older individual lived in 15 years ago, as an instrument for the accessibility of her *current* house. Column 3 of Table 2 shows the coefficients of interest of the instrumental variable specification including neighborhood fixed effects. Appendix B shows the complete regression results, including the control variables, and the results from the first stage regressions. Figure 6 presents the marginal effects. The results are similar to our main specification, with one exception: The marginal effect of living in a 0/3‐mix house for a 90 plus years old is now close to the marginal effect of living in a 2‐ or 3‐star house for someone of that age. Also, the standard deviations of the interaction terms have increased, which is often the case after using instruments (Baser, 2009). The time lag of 15 years is chosen because of data availability. Especially at higher ages, for instance, individuals who 15 years ago were already 75, the assumption that individuals at that age did not take their *current* expected health situation into account might be too strong. As an additional robustness check, we therefore rerun the IV analysis excluding all individuals who use home care at *t‐*1. The idea is that this only leaves cases who experience a new health shock in year *t.* We find similar results although the marginal effects of living in a 0/3‐mix house and living in a 3‐stars house are now almost the same size for persons aged 90 or older (results available upon request). Although selective moving might not be the only possible reason for endogeneity 21Another possible mechanism might be that older people living in an inaccessible house might be in better health, *because* they walk stairs every day. This health effect (if not captured by our health variables) might be a threat to our identification strategy, as we aim to estimate the effect of accessibility of the house on nursing home use in year *t*, *conditional* on health at the start of year *t.* This effect is not captured by our instrument., we interpret the IV results as supportive evidence for our main results.

## 6 CONCLUSION

So far, there has been no large scale study on the impact of the accessibility of the house an older person lives in on her use of nursing home care. This paper fills this gap. We have used unique population‐wide data to examine whether the accessibility of the house can postpone, or prevent, nursing home care use.

Our results provide some first evidence that the house should be added to the extensive list of determinants of nursing home care use found in earlier empirical research. We find that living in an accessible house is associated with a lower probability to use nursing home care. The relation increases with age, and the results are stronger for people with physical problems than for people with cognitive limitations. We also provide some evidence of substitution: older people in accessible house are more likely to use home care.

Our findings imply that there is indeed scope for policies that try to stimulate ageing in place through improvements in the living conditions of the older population. Because the effect of the house is concentrated at the highest age groups, policy makers should target the oldest old. They are most likely to benefit from improvements in the accessibility of the house. Large scale interventions targeting the total older population might be too general and therefore less likely to be (cost‐) effective.

We find that living in a house that cannot be made accessible leads to a higher likelihood of nursing home care use than living in a house that is or can be made accessible. In the Netherlands, the share of older people living in such houses is relatively small (Figure 2). However, in some neighborhoods, and possibly also in many other countries, this share is much larger. A complication is that policy interventions targeted at older people living in inadaptable houses would require either moving very old people to a more suitable house when their health already has deteriorated or motivating relatively young people to move to a more suitable house while they are still healthy. Both options seem hard to do.

However, we also find that living in a house that *can be made* accessible is equally beneficial, in terms of nursing home care use, as living in a house that actually *is* accessible. The share of older people living in such houses is much larger. If the equal effect is due to the fact that individuals living in adaptable house actually *do* adapt these houses, then our results also provide a motivation for (financially or otherwise) stimulating such adaptations. Unfortunately, we do not observe modifications that people make to their house, so that we cannot further support this conclusion. A relevant aspect in this regard is the relation between an individual's income and the accessibility of the house: it might be the case that subsidies are most effective when targeted at low income groups, as higher income groups might already be able to mitigate the effects of living in an inaccessible house. 22In an additional analysis suggested by one of the referees, we indeed find that the effects of accessibility on nursing home care use are driven by the effects for the low income groups.


It is important to take the context of the Dutch long‐term care system into account when generalizing our results to other institutional settings. First, the effects depend on the accessibility and funding for home care and nursing home care, which both are more generous in the Netherlands than in most other countries. The fact that the Dutch system is generous for both types of care is a good thing in terms of identification: our results are unlikely to be driven by differences in financial access to care. However, in other systems, where home care is less or more generously provided, the accessibility of the house might have a smaller or larger effect.

Second, during the time period of our analysis, housing conditions were officially taken into account in the assessment process to determine eligibility for nursing home care (although it is not clear to what extent they actually were in practice). We are not able to distinguish between the behavior of the older people themselves, who decide to apply for nursing home care eligibility, and the behavior of the assessors, who decide to grant eligibility. This means that our results could be partly driven by the eligibility criteria that were in place. 23Prior literature shows that not all people move to a nursing home immediately after obtaining the assessment (Bakx, Douven, & Schut, 2016). We performed a regression (not shown) with obtaining an assessment in year *t* as dependent variable. We find similar results as in our paper for moving to a nursing home in year *t*; hence, our results are not likely to be driven by persons postponing the move to a nursing home after obtaining an assessment.


A limitation of our study is that, with our data, we cannot disentangle the two channels behind the effect of the house on nursing home care use: the direct effect on health and the effect through coping. An area for future research would be to link our sample to data on hospital admissions and investigate whether living in an inaccessible house is related to more admissions for specific diagnoses related to a possible direct effect (e.g., falls and injuries).

## Supporting information

Data S1 Supporting Information
