# Supplementary material for: Can your house keep you out of a nursing home?
Source: Health Econ. 2020 Jan 31;29(5):540–53. doi: 10.1002/hec.4001 (PMC7187432; doi:10.1002/hec.4001)
Supplement: Supplementary file 1 — Data S1 Supporting Information [file HEC-29-540-s001.docx]

**Appendix A: Regression results to explain the use of nursing home care**

|  | 1 |
| --- | --- |
|  | Use of nursing home care |
| 0/3 mix | 0.054 |
|  | (0.085) |
| 2 stars | 0.126 |
|  | (0.079) |
| 3 stars | -0.009 |
|  | (0.080) |
| 0/3 mix * Age 80-84 | -0.253 |
|  | (0.156) |
| 0/3 mix * Age 85-89 | -0.777*** |
|  | (0.262) |
| 0/3 mix * Age 90 plus | -0.972* |
|  | (0.521) |
| 2 stars * Age 80-84 | -0.474*** |
|  | (0.143) |
| 2 stars * Age 85-89 | -1.242*** |
|  | (0.243) |
| 2 stars * Age 90 plus | -2.230*** |
|  | (0.488) |
| 3 stars * Age 80-84 | -0.352** |
|  | (0.145) |
| 3 stars * Age 85-89 | -0.992*** |
|  | (0.244) |
| 3 stars * Age 90 plus | -1.596*** |
|  | (0.485) |
| 2013 | 0.010 |
|  | (0.025) |
| 2014 | -0.258*** |
|  | (0.024) |
| Cholesterol reducer | -0.396*** |
|  | (0.023) |
| Diabetes | 0.201*** |
|  | (0.036) |
| Astma | -0.591*** |
|  | (0.030) |
| Antidepressants | 0.214*** |
|  | (0.047) |
| Antipsychotics | 6.217*** |
|  | (0.161) |
| Sleeping and tranquilizing tablets | -0.359*** |
|  | (0.065) |
| ADHD and nootropics | -0.638* |
|  | (0.370) |
| Other medicines | -1.397*** |
|  | (0.058) |
| Log of total GP care costs in euros | 0.761*** |
|  | (0.033) |
| Log of total pharmaceutical care costs in euros | 0.254*** |
|  | (0.011) |
| Log of total oral care costs in euros | -0.037*** |
|  | (0.005) |
| Log of total hospital care costs in euros | -0.106*** |
|  | (0.005) |
| Log of total paramedical care costs in euros | -0.047*** |
|  | (0.007) |
| Log of total technical aids costs in euros | 0.014*** |
|  | (0.004) |
| Log of total patient transport costs in euros | 0.017** |
|  | (0.007) |
| Log of total health care costs outside the Netherlands in euros | -0.100*** |
|  | (0.010) |
| Log of total other care costs in euros | -0.020*** |
|  | (0.005) |
| Gross income decile 2 | -0.079* |
|  | (0.042) |
| Gross income decile 3 | 0.021 |
|  | (0.044) |
| Gross income decile 4 | -0.062 |
|  | (0.045) |
| Gross income decile 5 | -0.118** |
|  | (0.047) |
| Gross income decile 6 | -0.160*** |
|  | (0.049) |
| Gross income decile 7 | -0.140*** |
|  | (0.053) |
| Gross income decile 8 | -0.197*** |
|  | (0.058) |
| Gross income decile 9 | -0.144** |
|  | (0.066) |
| Gross income decile 10 (highest) | -0.223*** |
|  | (0.075) |
| Financial wealth decile 2 | 0.037 |
|  | (0.052) |
| Financial wealth decile 3 | 0.061 |
|  | (0.052) |
| Financial wealth decile 4 | 0.169*** |
|  | (0.052) |
| Financial wealth decile 5 | 0.127** |
|  | (0.051) |
| Financial wealth decile 6 | 0.101** |
|  | (0.051) |
| Financial wealth decile 6 | 0.184*** |
|  | (0.051) |
| Financial wealth decile 8 | 0.166*** |
|  | (0.052) |
| Financial wealth decile 9 | 0.060 |
|  | (0.052) |
| Financial wealth decile 10 (highest) | -0.203*** |
|  | (0.054) |
| Male | 0.034 |
|  | (0.021) |
| Dutch | 0.525*** |
|  | (0.039) |
| Having a partner | -0.004 |
|  | (0.024) |
| Having children living at home | -0.261*** |
|  | (0.046) |
| Age 80-84 | 1.227*** |
|  | (0.141) |
| Age 85-89 | 3.469*** |
|  | (0.237) |
| Age 90plus | 6.598*** |
|  | (0.472) |
| Having children | -0.274*** |
|  | (0.034) |
| Home owner | -0.155*** |
|  | (0.024) |
| Living within 500 meter of supermarket | 0.028 |
|  | (0.027) |
| Within 500 meter of general practice | 0.004 |
|  | (0.030) |
| Living within 500 meter of general practic center | -0.220* |
|  | (0.133) |
| Living within 500 meter of pharmacy | 0.020 |
|  | (0.033) |
| Living within median distance to hospital | 0.052 |
|  | (0.059) |
| Domiciliary care | 0.621*** |
|  | (0.036) |
| Personal care | 4.143*** |
|  | (0.052) |
| Nursing | 0.198*** |
|  | (0.076) |
| Personal assistance | 7.718*** |
|  | (0.192) |
| Group assistance | 15.444*** |
|  | (0.173) |
| Constant | -3.028*** |
|  | (0.192) |
|  |  |
| Observations | 2,599,069 |
| R-squared | 0.079 |
| Neighborhood fixed effects | YES |
| Specification | OLS |

The coefficients are multiplied by 100 and hence express percentage points. **Appendix B: Results of the instrumental variable specification**

|  | 1 | 2 | 3 | 4 | 5 |
| --- | --- | --- | --- | --- | --- |
|  | 2 stars | 3 stars | 0/3 mix | 3 stars * Age 80-85 | 3 stars * Age 85-90 |
| 0/3 mix |  |  |  |  |  |
|  |  |  |  |  |  |
| 2 stars |  |  |  |  |  |
|  |  |  |  |  |  |
| 3 stars |  |  |  |  |  |
|  |  |  |  |  |  |
| 0/3 mix (15 years ago) | -2.135*** | -2.229*** | 54.708*** | 2.208*** | 1.161*** |
|  | (0.263) | (0.381) | (0.295) | (0.077) | (0.050) |
| 2 stars (15 years ago) | 56.007*** | -3.847*** | -1.993*** | 2.644*** | 1.218*** |
|  | (0.240) | (0.345) | (0.202) | (0.074) | (0.048) |
| 3 stars (15 years ago) | -9.719*** | 61.742*** | -1.819*** | 0.826*** | 0.466*** |
|  | (0.242) | (0.353) | (0.205) | (0.073) | (0.047) |
| 0/3 mix * Age 80-84 |  |  |  |  |  |
|  |  |  |  |  |  |
| 0/3 mix * Age 85-89 |  |  |  |  |  |
|  |  |  |  |  |  |
| 0/3 mix * Age 90 plus |  |  |  |  |  |
|  |  |  |  |  |  |
| 2 stars * Age 80-84 |  |  |  |  |  |
|  |  |  |  |  |  |
| 2 stars * Age 85-89 |  |  |  |  |  |
|  |  |  |  |  |  |
| 2 stars * Age 90 plus |  |  |  |  |  |
|  |  |  |  |  |  |
| 3 stars * Age 80-84 |  |  |  |  |  |
|  |  |  |  |  |  |
| 3 stars * Age 85-89 |  |  |  |  |  |
|  |  |  |  |  |  |
| 3 stars * Age 90 plus |  |  |  |  |  |
|  |  |  |  |  |  |
| 0/3 mix (15 years ago) * Age 80-84 | -0.189 | -2.377*** | 2.180*** | -11.687*** | 0.044 |
|  | (0.323) | (0.534) | (0.389) | (0.469) | (0.054) |
| 0/3 mix (15 years ago) * Age 85-89 | -0.283 | -2.685*** | 3.456*** | -0.178 | -11.485*** |
|  | (0.362) | (0.670) | (0.477) | (0.113) | (0.628) |
| 0/3 mix (15 years ago) * Age 90 plus | 0.120 | -2.745*** | 5.026*** | -0.670*** | -0.220** |
|  | (0.460) | (0.952) | (0.659) | (0.164) | (0.111) |
| 2 stars (15 years ago) * Age 80-84 | -1.163*** | -0.362 | 0.939*** | -12.068*** | 0.084* |
|  | (0.287) | (0.481) | (0.263) | (0.422) | (0.048) |
| 2 stars (15 years ago) * Age 85-89 | -2.454*** | 0.366 | 2.207*** | -0.248** | -10.583*** |
|  | (0.332) | (0.604) | (0.318) | (0.101) | (0.566) |
| 2 stars (15 years ago) * Age 90 plus | -1.579*** | 0.584 | 2.751*** | -0.757*** | -0.353*** |
|  | (0.452) | (0.871) | (0.451) | (0.149) | (0.101) |
| 3 stars (15 years ago) * Age 80-84 | 2.207*** | -3.051*** | 0.303 | 57.676*** | -0.278*** |
|  | (0.294) | (0.492) | (0.268) | (0.424) | (0.050) |
| 3 stars (15 years ago) * Age 85-89 | 4.629*** | -5.515*** | 1.051*** | -1.514*** | 57.727*** |
|  | (0.330) | (0.612) | (0.323) | (0.104) | (0.562) |
| 3 stars (15 years ago) * Age 90 plus | 6.949*** | -6.761*** | 1.587*** | -2.810*** | -1.771*** |
|  | (0.425) | (0.871) | (0.452) | (0.153) | (0.103) |
| 2013 | 0.111*** | -0.132*** | 0.008 | -0.037* | -0.010 |
|  | (0.024) | (0.024) | (0.016) | (0.019) | (0.014) |
| 2014 | 0.176*** | -0.211*** | 0.012 | -0.057** | 0.009 |
|  | (0.032) | (0.032) | (0.021) | (0.025) | (0.018) |
| Cholestorol reducer | -0.343*** | 0.362*** | 0.009 | 0.103** | -0.002 |
|  | (0.076) | (0.077) | (0.050) | (0.045) | (0.032) |
| Diabetes | 0.499*** | -0.377*** | -0.083 | -0.148** | -0.087* |
|  | (0.111) | (0.114) | (0.075) | (0.067) | (0.045) |
| Astma | 0.367*** | -0.439*** | 0.027 | -0.176*** | -0.122*** |
|  | (0.091) | (0.093) | (0.062) | (0.055) | (0.038) |
| Antidepressants | -0.857*** | 0.657*** | 0.145* | 0.284*** | 0.006 |
|  | (0.118) | (0.121) | (0.080) | (0.072) | (0.050) |
| Antipsychotics | 0.813*** | -0.770*** | -0.085 | -0.313** | -0.274*** |
|  | (0.222) | (0.229) | (0.153) | (0.137) | (0.104) |
| Sleeping and tranquilizing tablets | -0.094 | 0.138 | -0.014 | 0.156* | -0.043 |
|  | (0.144) | (0.152) | (0.105) | (0.091) | (0.067) |
| ADHD and nootropics | -1.257 | -0.305 | 0.825 | -0.460 | -0.602 |
|  | (0.957) | (0.972) | (0.667) | (0.550) | (0.445) |
| Other medicines | 0.353** | -0.012 | -0.274** | -0.182* | -0.056 |
|  | (0.174) | (0.175) | (0.114) | (0.099) | (0.067) |
| Log of total GP care costs in euros | -0.609*** | 0.517*** | 0.112** | 0.171*** | 0.119*** |
|  | (0.068) | (0.069) | (0.046) | (0.041) | (0.031) |
| Log of total pharmaceutical care costs in euros | -0.383*** | 0.299*** | 0.087*** | 0.106*** | 0.024* |
|  | (0.032) | (0.032) | (0.021) | (0.019) | (0.013) |
| Log of total oral care costs in euros | -0.095*** | 0.077*** | 0.021** | 0.028*** | 0.016*** |
|  | (0.014) | (0.015) | (0.009) | (0.009) | (0.006) |
| Log of total hospital care costs in euros | -0.062*** | 0.068*** | 0.003 | 0.013* | 0.006 |
|  | (0.012) | (0.012) | (0.008) | (0.007) | (0.005) |
| Log of total paramedical care costs in euros | -0.036** | 0.051*** | -0.001 | 0.008 | -0.018** |
|  | (0.016) | (0.017) | (0.011) | (0.010) | (0.007) |
| Log of total technical aids costs in euros | -0.142*** | 0.143*** | 0.009 | 0.066*** | 0.009* |
|  | (0.011) | (0.011) | (0.007) | (0.007) | (0.005) |
| Log of total patient transport costs in euros | 0.095*** | -0.103*** | 0.002 | -0.038*** | -0.036*** |
|  | (0.013) | (0.014) | (0.009) | (0.008) | (0.006) |
| Log of total health care costs outside the Netherlands in euros | -0.082** | -0.026 | 0.060** | -0.001 | -0.004 |
|  | (0.036) | (0.037) | (0.026) | (0.020) | (0.011) |
| Log of total other care costs in euros | -0.085*** | 0.057*** | 0.022** | 0.010 | 0.017** |
|  | (0.016) | (0.017) | (0.011) | (0.010) | (0.007) |
| Gross income decile 2 | -0.537*** | 1.905*** | -0.964*** | 0.640*** | 0.613*** |
|  | (0.115) | (0.120) | (0.087) | (0.071) | (0.055) |
| Gross income decile 3 | 0.483*** | 1.313*** | -1.334*** | 0.474*** | 0.368*** |
|  | (0.129) | (0.132) | (0.093) | (0.077) | (0.058) |
| Gross income decile 4 | 0.097 | 1.926*** | -1.439*** | 0.754*** | 0.440*** |
|  | (0.139) | (0.142) | (0.098) | (0.082) | (0.061) |
| Gross income decile 5 | 0.010 | 2.179*** | -1.552*** | 0.769*** | 0.517*** |
|  | (0.148) | (0.152) | (0.102) | (0.087) | (0.064) |
| Gross income decile 6 | -0.097 | 2.183*** | -1.482*** | 0.767*** | 0.628*** |
|  | (0.162) | (0.164) | (0.108) | (0.093) | (0.068) |
| Gross income decile 7 | 0.500*** | 2.049*** | -1.742*** | 0.803*** | 0.595*** |
|  | (0.177) | (0.179) | (0.116) | (0.101) | (0.073) |
| Gross income decile 8 | 1.249*** | 1.621*** | -1.928*** | 0.657*** | 0.652*** |
|  | (0.200) | (0.200) | (0.128) | (0.112) | (0.081) |
| Gross income decile 9 | 1.496*** | 1.622*** | -2.007*** | 0.721*** | 0.594*** |
|  | (0.231) | (0.228) | (0.147) | (0.127) | (0.090) |
| Gross income decile 10 (highest) | 2.871*** | 0.541** | -2.025*** | 0.494*** | 0.455*** |
|  | (0.279) | (0.270) | (0.179) | (0.148) | (0.110) |
| Financial wealth decile 2 | -0.307** | 1.294*** | -0.609*** | 0.318*** | 0.015 |
|  | (0.147) | (0.152) | (0.111) | (0.085) | (0.056) |
| Financial wealth decile 3 | -0.986*** | 1.782*** | -0.393*** | 0.652*** | 0.204*** |
|  | (0.152) | (0.156) | (0.111) | (0.087) | (0.058) |
| Financial wealth decile 4 | -1.138*** | 1.753*** | -0.189* | 0.635*** | 0.167*** |
|  | (0.153) | (0.156) | (0.111) | (0.087) | (0.058) |
| Financial wealth decile 5 | -1.165*** | 1.674*** | -0.133 | 0.594*** | 0.202*** |
|  | (0.152) | (0.155) | (0.109) | (0.086) | (0.059) |
| Financial wealth decile 6 | -1.806*** | 2.168*** | 0.021 | 0.779*** | 0.317*** |
|  | (0.157) | (0.160) | (0.110) | (0.089) | (0.060) |
| Financial wealth decile 6 | -2.797*** | 2.900*** | 0.133 | 0.988*** | 0.308*** |
|  | (0.159) | (0.161) | (0.111) | (0.090) | (0.060) |
| Financial wealth decile 8 | -4.687*** | 4.373*** | 0.550*** | 1.464*** | 0.520*** |
|  | (0.167) | (0.169) | (0.115) | (0.095) | (0.064) |
| Financial wealth decile 9 | -6.351*** | 5.515*** | 1.045*** | 1.805*** | 0.733*** |
|  | (0.171) | (0.173) | (0.118) | (0.097) | (0.066) |
| Financial wealth decile 10 (highest) | -5.811*** | 5.135*** | 0.856*** | 1.720*** | 0.751*** |
|  | (0.185) | (0.185) | (0.124) | (0.103) | (0.071) |
| Male | 0.686*** | -0.705*** | -0.047 | -0.193*** | -0.001 |
|  | (0.076) | (0.076) | (0.049) | (0.043) | (0.029) |
| Dutch | -2.750*** | 2.898*** | 0.295*** | 0.757*** | 0.282*** |
|  | (0.135) | (0.141) | (0.099) | (0.078) | (0.057) |
| Having a partner | 0.417*** | 0.127 | -0.529*** | 0.005 | 0.227*** |
|  | (0.086) | (0.087) | (0.056) | (0.050) | (0.034) |
| Having children living at home | 10.261*** | -9.129*** | -1.552*** | -2.921*** | -1.871*** |
|  | (0.153) | (0.146) | (0.092) | (0.079) | (0.057) |
| Age 80-84 | -0.614** | 1.846*** | -0.650** | 33.164*** | -0.468*** |
|  | (0.271) | (0.473) | (0.258) | (0.414) | (0.047) |
| Age 85-89 | -0.497 | 2.301*** | -1.831*** | -1.077*** | 34.464*** |
|  | (0.304) | (0.591) | (0.311) | (0.100) | (0.552) |
| Age 90plus | -0.348 | 1.683** | -2.915*** | -0.817*** | -0.696*** |
|  | (0.393) | (0.846) | (0.436) | (0.147) | (0.100) |
| Having children | -2.286*** | 2.093*** | 0.426*** | 0.836*** | 0.511*** |
|  | (0.101) | (0.107) | (0.073) | (0.060) | (0.045) |
| Home owner | 13.183*** | -9.464*** | -3.543*** | -3.263*** | -1.863*** |
|  | (0.090) | (0.088) | (0.056) | (0.049) | (0.033) |
| Living within 500 meter of supermarket | -2.939*** | 1.004*** | 1.610*** | 0.369*** | 0.096*** |
|  | (0.091) | (0.092) | (0.061) | (0.052) | (0.036) |
| Within 500 meter of general practice | -1.606*** | 0.926*** | 0.626*** | 0.276*** | 0.256*** |
|  | (0.100) | (0.102) | (0.068) | (0.058) | (0.040) |
| Living within 500 meter of general practic center | -1.168** | 0.896* | 0.248 | 0.203 | -0.229 |
|  | (0.464) | (0.492) | (0.328) | (0.277) | (0.193) |
| Living within 500 meter of pharmacy | -2.437*** | 1.447*** | 0.854*** | 0.525*** | 0.171*** |
|  | (0.108) | (0.111) | (0.076) | (0.064) | (0.044) |
| Living within median distance to hospital | 0.608*** | -1.119*** | 0.328*** | -0.444*** | -0.315*** |
|  | (0.168) | (0.172) | (0.110) | (0.099) | (0.069) |
| Domiciliary care in prior year | -2.016*** | 2.353*** | -0.019 | 0.990*** | 0.465*** |
|  | (0.094) | (0.098) | (0.066) | (0.060) | (0.047) |
| Personal care in prior year | -3.027*** | 2.920*** | 0.279*** | 1.105*** | 1.011*** |
|  | (0.100) | (0.104) | (0.071) | (0.065) | (0.056) |
| Nursing in prior year | 0.845*** | -0.666*** | -0.207** | -0.361*** | -0.043 |
|  | (0.120) | (0.125) | (0.084) | (0.078) | (0.069) |
| Personal assistance in prior year | 0.813*** | -0.338 | -0.302* | -0.278* | -0.102 |
|  | (0.228) | (0.246) | (0.174) | (0.147) | (0.138) |
| Group assistance in prior year | -2.259*** | 2.154*** | 0.251 | 0.772*** | 0.426*** |
|  | (0.211) | (0.222) | (0.155) | (0.137) | (0.121) |
|  |  |  |  |  |  |
| Observations | 2,466,684 | 2,466,684 | 2,466,684 | 2,466,684 | 2,466,684 |
| R-squared | 0.557 | 0.515 | 0.478 | 0.598 | 0.651 |
| Neighborhood fixed effects | YES | YES | YES | YES | YES |

|  | 6 | 7 | 8 | 9 | 10 |
| --- | --- | --- | --- | --- | --- |
|  | 3 stars * Age 90 plus | 2 stars * Age 80-85 | 2 stars * Age 85-90 | 2 stars * Age 90 plus | 0/3 mix * Age 80-85 |
| 0/3 mix |  |  |  |  |  |
|  |  |  |  |  |  |
| 2 stars |  |  |  |  |  |
|  |  |  |  |  |  |
| 3 stars |  |  |  |  |  |
|  |  |  |  |  |  |
| 0/3 mix (15 years ago) | 0.450*** | -0.863*** | -0.429*** | -0.156*** | -2.640*** |
|  | (0.026) | (0.060) | (0.036) | (0.017) | (0.052) |
| 2 stars (15 years ago) | 0.384*** | -4.169*** | -1.825*** | -0.566*** | -0.112** |
|  | (0.025) | (0.060) | (0.037) | (0.019) | (0.044) |
| 3 stars (15 years ago) | 0.159*** | -2.792*** | -1.327*** | -0.428*** | 0.292*** |
|  | (0.025) | (0.061) | (0.037) | (0.018) | (0.043) |
| 0/3 mix * Age 80-84 |  |  |  |  |  |
|  |  |  |  |  |  |
| 0/3 mix * Age 85-89 |  |  |  |  |  |
|  |  |  |  |  |  |
| 0/3 mix * Age 90 plus |  |  |  |  |  |
|  |  |  |  |  |  |
| 2 stars * Age 80-84 |  |  |  |  |  |
|  |  |  |  |  |  |
| 2 stars * Age 85-89 |  |  |  |  |  |
|  |  |  |  |  |  |
| 2 stars * Age 90 plus |  |  |  |  |  |
|  |  |  |  |  |  |
| 3 stars * Age 80-84 |  |  |  |  |  |
|  |  |  |  |  |  |
| 3 stars * Age 85-89 |  |  |  |  |  |
|  |  |  |  |  |  |
| 3 stars * Age 90 plus |  |  |  |  |  |
|  |  |  |  |  |  |
| 0/3 mix (15 years ago) * Age 80-84 | 0.012 | 0.565** | -0.051 | -0.030* | 65.512*** |
|  | (0.024) | (0.220) | (0.038) | (0.016) | (0.322) |
| 0/3 mix (15 years ago) * Age 85-89 | 0.028 | 0.083 | 0.201 | 0.010 | -0.215*** |
|  | (0.035) | (0.085) | (0.223) | (0.022) | (0.067) |
| 0/3 mix (15 years ago) * Age 90 plus | -10.393*** | 0.312** | 0.179** | -0.015 | -0.374*** |
|  | (1.022) | (0.123) | (0.076) | (0.308) | (0.097) |
| 2 stars (15 years ago) * Age 80-84 | 0.038* | 68.100*** | -0.083** | -0.037*** | -0.967*** |
|  | (0.021) | (0.204) | (0.034) | (0.014) | (0.212) |
| 2 stars (15 years ago) * Age 85-89 | 0.047 | 0.117 | 66.325*** | -0.044** | -0.137** |
|  | (0.031) | (0.075) | (0.235) | (0.020) | (0.054) |
| 2 stars (15 years ago) * Age 90 plus | -9.093*** | 0.159 | -0.018 | 67.126*** | -0.091 |
|  | (0.939) | (0.112) | (0.070) | (0.376) | (0.080) |
| 3 stars (15 years ago) * Age 80-84 | -0.075*** | 0.270 | 0.231*** | 0.055*** | -2.693*** |
|  | (0.022) | (0.197) | (0.036) | (0.015) | (0.215) |
| 3 stars (15 years ago) * Age 85-89 | -0.237*** | 1.238*** | 0.602*** | 0.191*** | -0.013 |
|  | (0.032) | (0.080) | (0.202) | (0.021) | (0.055) |
| 3 stars (15 years ago) * Age 90 plus | 60.445*** | 2.021*** | 1.209*** | 0.285 | 0.071 |
|  | (0.921) | (0.116) | (0.072) | (0.287) | (0.081) |
| 2013 | -0.006 | 0.058*** | -0.003 | -0.004 | -0.030** |
|  | (0.008) | (0.019) | (0.013) | (0.007) | (0.012) |
| 2014 | -0.011 | 0.082*** | -0.023 | -0.008 | -0.027* |
|  | (0.010) | (0.025) | (0.017) | (0.009) | (0.016) |
| Cholestorol reducer | -0.028* | -0.074* | 0.003 | 0.034** | -0.016 |
|  | (0.016) | (0.044) | (0.030) | (0.015) | (0.029) |
| Diabetes | -0.094*** | 0.212*** | 0.104** | 0.103*** | -0.035 |
|  | (0.024) | (0.065) | (0.042) | (0.021) | (0.042) |
| Astma | -0.071*** | 0.143*** | 0.098*** | 0.066*** | 0.037 |
|  | (0.022) | (0.053) | (0.036) | (0.020) | (0.035) |
| Antidepressants | -0.018 | -0.349*** | -0.062 | 0.055** | 0.048 |
|  | (0.028) | (0.070) | (0.047) | (0.025) | (0.046) |
| Antipsychotics | -0.317*** | 0.347*** | 0.311*** | 0.341*** | -0.074 |
|  | (0.070) | (0.131) | (0.099) | (0.064) | (0.087) |
| Sleeping and tranquilizing tablets | 0.007 | -0.109 | 0.054 | -0.002 | -0.000 |
|  | (0.042) | (0.086) | (0.061) | (0.036) | (0.060) |
| ADHD and nootropics | 0.288 | -0.148 | 0.041 | -0.229 | 0.634 |
|  | (0.292) | (0.534) | (0.418) | (0.268) | (0.390) |
| Other medicines | -0.106*** | 0.244** | 0.127** | 0.108*** | -0.035 |
|  | (0.039) | (0.098) | (0.064) | (0.035) | (0.062) |
| Log of total GP care costs in euros | -0.021 | -0.213*** | -0.133*** | -0.011 | 0.046* |
|  | (0.021) | (0.040) | (0.030) | (0.019) | (0.027) |
| Log of total pharmaceutical care costs in euros | 0.034*** | -0.125*** | -0.037*** | -0.040*** | 0.016 |
|  | (0.008) | (0.018) | (0.012) | (0.007) | (0.012) |
| Log of total oral care costs in euros | -0.001 | -0.035*** | -0.017*** | -0.001 | 0.005 |
|  | (0.003) | (0.009) | (0.005) | (0.003) | (0.005) |
| Log of total hospital care costs in euros | 0.008** | -0.007 | -0.005 | -0.004 | -0.005 |
|  | (0.003) | (0.007) | (0.005) | (0.003) | (0.005) |
| Log of total paramedical care costs in euros | -0.027*** | -0.004 | 0.025*** | 0.024*** | 0.002 |
|  | (0.004) | (0.010) | (0.007) | (0.004) | (0.007) |
| Log of total technical aids costs in euros | -0.009*** | -0.062*** | -0.010** | 0.010*** | 0.001 |
|  | (0.003) | (0.006) | (0.004) | (0.002) | (0.004) |
| Log of total patient transport costs in euros | -0.015*** | 0.031*** | 0.036*** | 0.018*** | 0.003 |
|  | (0.004) | (0.008) | (0.006) | (0.003) | (0.005) |
| Log of total health care costs outside the Netherlands in euros | 0.009 | -0.031 | -0.008 | -0.004 | 0.016 |
|  | (0.006) | (0.020) | (0.011) | (0.006) | (0.014) |
| Log of total other care costs in euros | 0.009** | -0.030*** | -0.016** | -0.009*** | 0.017*** |
|  | (0.004) | (0.010) | (0.006) | (0.003) | (0.006) |
| Gross income decile 2 | 0.311*** | -0.226*** | -0.320*** | -0.110*** | -0.327*** |
|  | (0.036) | (0.067) | (0.051) | (0.031) | (0.049) |
| Gross income decile 3 | 0.218*** | 0.044 | 0.006 | -0.009 | -0.392*** |
|  | (0.036) | (0.075) | (0.054) | (0.032) | (0.052) |
| Gross income decile 4 | 0.166*** | -0.144* | -0.103* | 0.003 | -0.478*** |
|  | (0.038) | (0.080) | (0.057) | (0.034) | (0.055) |
| Gross income decile 5 | 0.199*** | -0.094 | -0.142** | -0.032 | -0.524*** |
|  | (0.039) | (0.085) | (0.060) | (0.036) | (0.057) |
| Gross income decile 6 | 0.202*** | -0.187** | -0.231*** | -0.034 | -0.413*** |
|  | (0.041) | (0.092) | (0.065) | (0.038) | (0.060) |
| Gross income decile 7 | 0.220*** | -0.075 | -0.201*** | -0.023 | -0.527*** |
|  | (0.044) | (0.100) | (0.070) | (0.041) | (0.064) |
| Gross income decile 8 | 0.240*** | 0.183 | -0.202** | -0.025 | -0.560*** |
|  | (0.049) | (0.112) | (0.078) | (0.047) | (0.070) |
| Gross income decile 9 | 0.278*** | 0.189 | -0.065 | -0.061 | -0.637*** |
|  | (0.055) | (0.129) | (0.090) | (0.053) | (0.081) |
| Gross income decile 10 (highest) | 0.086 | 0.348** | 0.142 | 0.222*** | -0.465*** |
|  | (0.067) | (0.155) | (0.112) | (0.070) | (0.097) |
| Financial wealth decile 2 | 0.042 | -0.015 | 0.002 | -0.045 | -0.192*** |
|  | (0.032) | (0.081) | (0.050) | (0.027) | (0.060) |
| Financial wealth decile 3 | 0.049 | -0.481*** | -0.167*** | -0.061** | -0.091 |
|  | (0.034) | (0.084) | (0.053) | (0.029) | (0.061) |
| Financial wealth decile 4 | 0.087** | -0.430*** | -0.228*** | -0.119*** | -0.098 |
|  | (0.034) | (0.084) | (0.053) | (0.030) | (0.060) |
| Financial wealth decile 5 | 0.090*** | -0.394*** | -0.276*** | -0.154*** | -0.097 |
|  | (0.035) | (0.084) | (0.054) | (0.031) | (0.059) |
| Financial wealth decile 6 | 0.114*** | -0.658*** | -0.418*** | -0.162*** | -0.025 |
|  | (0.034) | (0.087) | (0.056) | (0.031) | (0.060) |
| Financial wealth decile 6 | 0.115*** | -0.909*** | -0.394*** | -0.175*** | -0.060 |
|  | (0.035) | (0.088) | (0.057) | (0.031) | (0.060) |
| Financial wealth decile 8 | 0.239*** | -1.521*** | -0.684*** | -0.304*** | 0.113* |
|  | (0.037) | (0.093) | (0.060) | (0.033) | (0.063) |
| Financial wealth decile 9 | 0.279*** | -2.022*** | -1.036*** | -0.394*** | 0.258*** |
|  | (0.038) | (0.095) | (0.063) | (0.035) | (0.064) |
| Financial wealth decile 10 (highest) | 0.206*** | -1.900*** | -0.958*** | -0.315*** | 0.240*** |
|  | (0.043) | (0.102) | (0.068) | (0.040) | (0.068) |
| Male | 0.033** | 0.278*** | 0.023 | -0.039*** | -0.089*** |
|  | (0.016) | (0.042) | (0.028) | (0.015) | (0.027) |
| Dutch | 0.131*** | -0.741*** | -0.384*** | -0.116*** | 0.081 |
|  | (0.034) | (0.075) | (0.052) | (0.030) | (0.053) |
| Having a partner | 0.125*** | 0.103** | -0.263*** | -0.160*** | -0.111*** |
|  | (0.018) | (0.050) | (0.033) | (0.017) | (0.031) |
| Having children living at home | -0.768*** | 3.395*** | 2.161*** | 0.868*** | -0.594*** |
|  | (0.036) | (0.084) | (0.061) | (0.038) | (0.048) |
| Age 80-84 | -0.186*** | 5.264*** | 0.531*** | 0.200*** | 5.869*** |
|  | (0.022) | (0.184) | (0.033) | (0.014) | (0.207) |
| Age 85-89 | -0.353*** | 1.585*** | 3.603*** | 0.372*** | -0.213*** |
|  | (0.032) | (0.074) | (0.190) | (0.021) | (0.054) |
| Age 90plus | 33.485*** | 1.938*** | 1.210*** | 2.406*** | -0.403*** |
|  | (0.908) | (0.109) | (0.069) | (0.273) | (0.080) |
| Having children | 0.180*** | -0.902*** | -0.603*** | -0.205*** | 0.144*** |
|  | (0.028) | (0.056) | (0.041) | (0.024) | (0.040) |
| Home owner | -0.674*** | 4.443*** | 2.536*** | 0.905*** | -1.123*** |
|  | (0.018) | (0.050) | (0.033) | (0.018) | (0.031) |
| Living within 500 meter of supermarket | -0.024 | -1.053*** | -0.388*** | -0.054*** | 0.584*** |
|  | (0.020) | (0.051) | (0.034) | (0.019) | (0.033) |
| Within 500 meter of general practice | 0.110*** | -0.489*** | -0.330*** | -0.119*** | 0.193*** |
|  | (0.023) | (0.057) | (0.038) | (0.021) | (0.038) |
| Living within 500 meter of general practic center | -0.054 | -0.099 | 0.195 | 0.078 | -0.035 |
|  | (0.113) | (0.262) | (0.174) | (0.100) | (0.179) |
| Living within 500 meter of pharmacy | 0.018 | -0.874*** | -0.357*** | -0.049** | 0.310*** |
|  | (0.026) | (0.062) | (0.042) | (0.023) | (0.042) |
| Living within median distance to hospital | -0.190*** | 0.260*** | 0.192*** | 0.101*** | 0.151** |
|  | (0.039) | (0.095) | (0.064) | (0.035) | (0.062) |
| Domiciliary care in prior year | 0.161*** | -0.919*** | -0.426*** | -0.151*** | 0.042 |
|  | (0.028) | (0.058) | (0.044) | (0.025) | (0.039) |
| Personal care in prior year | 0.470*** | -1.207*** | -1.002*** | -0.488*** | 0.190*** |
|  | (0.036) | (0.062) | (0.052) | (0.033) | (0.042) |
| Nursing in prior year | -0.181*** | 0.406*** | 0.091 | 0.269*** | -0.051 |
|  | (0.048) | (0.074) | (0.064) | (0.043) | (0.050) |
| Personal assistance in prior year | 0.302*** | 0.532*** | 0.073 | -0.258*** | -0.245** |
|  | (0.114) | (0.136) | (0.125) | (0.100) | (0.097) |
| Group assistance in prior year | 0.231*** | -0.814*** | -0.530*** | -0.239*** | 0.035 |
|  | (0.083) | (0.131) | (0.112) | (0.074) | (0.090) |
|  |  |  |  |  |  |
| Observations | 2,466,684 | 2,466,684 | 2,466,684 | 2,466,684 | 2,466,684 |
| R-squared | 0.705 | 0.677 | 0.669 | 0.678 | 0.457 |
| Neighborhood fixed effects | YES | YES | YES | YES | YES |

|  | 11 | 12 | 13 |
| --- | --- | --- | --- |
|  | 0/3 mix * Age 85-90 | 0/3 mix * Age 90 plus | Use of nursing home care |
| 0/3 mix |  |  | 0.292* |
|  |  |  | (0.149) |
| 2 stars |  |  | 0.399*** |
|  |  |  | (0.136) |
| 3 stars |  |  | 0.389*** |
|  |  |  | (0.138) |
| 0/3 mix (15 years ago) | -1.318*** | -0.486*** |  |
|  | (0.034) | (0.019) |  |
| 2 stars (15 years ago) | -0.150*** | -0.073*** |  |
|  | (0.029) | (0.016) |  |
| 3 stars (15 years ago) | 0.083*** | 0.011 |  |
|  | (0.028) | (0.015) |  |
| 0/3 mix * Age 80-84 |  |  | -0.407 |
|  |  |  | (0.261) |
| 0/3 mix * Age 85-89 |  |  | -0.717* |
|  |  |  | (0.409) |
| 0/3 mix * Age 90 plus |  |  | -1.803** |
|  |  |  | (0.780) |
| 2 stars * Age 80-84 |  |  | -0.766*** |
|  |  |  | (0.233) |
| 2 stars * Age 85-89 |  |  | -1.494*** |
|  |  |  | (0.368) |
| 2 stars * Age 90 plus |  |  | -3.680*** |
|  |  |  | (0.719) |
| 3 stars * Age 80-84 |  |  | -0.515** |
|  |  |  | (0.242) |
| 3 stars * Age 85-89 |  |  | -0.770** |
|  |  |  | (0.382) |
| 3 stars * Age 90 plus |  |  | -2.438*** |
|  |  |  | (0.737) |
| 0/3 mix (15 years ago) * Age 80-84 | -0.089*** | -0.026* |  |
|  | (0.031) | (0.014) |  |
| 0/3 mix (15 years ago) * Age 85-89 | 66.955*** | -0.102*** |  |
|  | (0.414) | (0.021) |  |
| 0/3 mix (15 years ago) * Age 90 plus | -0.449*** | 69.066*** |  |
|  | (0.063) | (0.638) |  |
| 2 stars (15 years ago) * Age 80-84 | -0.084*** | -0.038*** |  |
|  | (0.025) | (0.011) |  |
| 2 stars (15 years ago) * Age 85-89 | 0.371 | -0.051*** |  |
|  | (0.267) | (0.017) |  |
| 2 stars (15 years ago) * Age 90 plus | -0.097* | 0.764* |  |
|  | (0.052) | (0.425) |  |
| 3 stars (15 years ago) * Age 80-84 | -0.046* | -0.023** |  |
|  | (0.026) | (0.012) |  |
| 3 stars (15 years ago) * Age 85-89 | -2.008*** | -0.020 |  |
|  | (0.269) | (0.017) |  |
| 3 stars (15 years ago) * Age 90 plus | 0.051 | -1.734*** |  |
|  | (0.053) | (0.421) |  |
| 2013 | 0.010 | 0.012** | 0.013 |
|  | (0.009) | (0.005) | (0.026) |
| 2014 | 0.007 | 0.019*** | -0.255*** |
|  | (0.012) | (0.007) | (0.025) |
| Cholestorol reducer | -0.005 | -0.007 | -0.391*** |
|  | (0.020) | (0.011) | (0.023) |
| Diabetes | -0.013 | 0.001 | 0.202*** |
|  | (0.029) | (0.016) | (0.037) |
| Astma | 0.017 | -0.005 | -0.596*** |
|  | (0.025) | (0.015) | (0.031) |
| Antidepressants | 0.036 | -0.036* | 0.213*** |
|  | (0.033) | (0.019) | (0.048) |
| Antipsychotics | -0.044 | -0.039 | 6.253*** |
|  | (0.068) | (0.045) | (0.166) |
| Sleeping and tranquilizing tablets | -0.036 | -0.001 | -0.360*** |
|  | (0.045) | (0.028) | (0.067) |
| ADHD and nootropics | 0.181 | -0.118 | -0.645* |
|  | (0.316) | (0.192) | (0.383) |
| Other medicines | -0.052 | -0.005 | -1.392*** |
|  | (0.043) | (0.026) | (0.059) |
| Log of total GP care costs in euros | 0.020 | 0.028** | 0.802*** |
|  | (0.019) | (0.014) | (0.034) |
| Log of total pharmaceutical care costs in euros | 0.012 | 0.007 | 0.255*** |
|  | (0.008) | (0.005) | (0.011) |
| Log of total oral care costs in euros | 0.002 | 0.001 | -0.037*** |
|  | (0.004) | (0.002) | (0.006) |
| Log of total hospital care costs in euros | -0.000 | -0.001 | -0.108*** |
|  | (0.003) | (0.002) | (0.005) |
| Log of total paramedical care costs in euros | -0.008* | 0.001 | -0.047*** |
|  | (0.005) | (0.003) | (0.008) |
| Log of total technical aids costs in euros | 0.002 | -0.001 | 0.012*** |
|  | (0.003) | (0.002) | (0.004) |
| Log of total patient transport costs in euros | -0.001 | -0.003 | 0.014* |
|  | (0.004) | (0.002) | (0.007) |
| Log of total health care costs outside the Netherlands in euros | 0.008 | -0.004 | -0.100*** |
|  | (0.009) | (0.004) | (0.010) |
| Log of total other care costs in euros | 0.002 | -0.002 | -0.020*** |
|  | (0.004) | (0.003) | (0.006) |
| Gross income decile 2 | -0.185*** | -0.164*** | -0.074* |
|  | (0.039) | (0.025) | (0.044) |
| Gross income decile 3 | -0.274*** | -0.168*** | 0.003 |
|  | (0.039) | (0.025) | (0.045) |
| Gross income decile 4 | -0.233*** | -0.143*** | -0.070 |
|  | (0.041) | (0.026) | (0.047) |
| Gross income decile 5 | -0.256*** | -0.137*** | -0.134*** |
|  | (0.042) | (0.027) | (0.049) |
| Gross income decile 6 | -0.279*** | -0.150*** | -0.179*** |
|  | (0.044) | (0.027) | (0.051) |
| Gross income decile 7 | -0.261*** | -0.170*** | -0.167*** |
|  | (0.047) | (0.029) | (0.055) |
| Gross income decile 8 | -0.304*** | -0.171*** | -0.246*** |
|  | (0.050) | (0.032) | (0.060) |
| Gross income decile 9 | -0.313*** | -0.154*** | -0.176*** |
|  | (0.056) | (0.035) | (0.068) |
| Gross income decile 10 (highest) | -0.354*** | -0.229*** | -0.271*** |
|  | (0.067) | (0.041) | (0.078) |
| Financial wealth decile 2 | 0.012 | 0.016 | 0.040 |
|  | (0.040) | (0.024) | (0.054) |
| Financial wealth decile 3 | 0.005 | 0.023 | 0.031 |
|  | (0.040) | (0.024) | (0.054) |
| Financial wealth decile 4 | 0.081** | 0.047* | 0.142*** |
|  | (0.040) | (0.024) | (0.054) |
| Financial wealth decile 5 | 0.083** | 0.067*** | 0.118** |
|  | (0.040) | (0.024) | (0.053) |
| Financial wealth decile 6 | 0.113*** | 0.057** | 0.080 |
|  | (0.040) | (0.024) | (0.053) |
| Financial wealth decile 6 | 0.095** | 0.060** | 0.174*** |
|  | (0.040) | (0.024) | (0.053) |
| Financial wealth decile 8 | 0.130*** | 0.054** | 0.168*** |
|  | (0.042) | (0.025) | (0.054) |
| Financial wealth decile 9 | 0.268*** | 0.105*** | 0.043 |
|  | (0.043) | (0.026) | (0.054) |
| Financial wealth decile 10 (highest) | 0.147*** | 0.074*** | -0.215*** |
|  | (0.046) | (0.027) | (0.056) |
| Male | -0.023 | 0.007 | 0.028 |
|  | (0.018) | (0.010) | (0.021) |
| Dutch | 0.123*** | -0.006 | 0.480*** |
|  | (0.037) | (0.024) | (0.041) |
| Having a partner | 0.020 | 0.040*** | 0.016 |
|  | (0.021) | (0.011) | (0.025) |
| Having children living at home | -0.340*** | -0.122*** | -0.205*** |
|  | (0.033) | (0.021) | (0.048) |
| Age 80-84 | 0.017 | 0.020* | 1.451*** |
|  | (0.025) | (0.011) | (0.230) |
| Age 85-89 | 5.189*** | 0.025 | 3.470*** |
|  | (0.258) | (0.018) | (0.362) |
| Age 90plus | -0.068 | 4.730*** | 7.662*** |
|  | (0.052) | (0.406) | (0.701) |
| Having children | 0.145*** | 0.035* | -0.282*** |
|  | (0.030) | (0.019) | (0.035) |
| Home owner | -0.647*** | -0.235*** | -0.127*** |
|  | (0.021) | (0.012) | (0.025) |
| Living within 500 meter of supermarket | 0.235*** | 0.059*** | 0.032 |
|  | (0.023) | (0.014) | (0.027) |
| Within 500 meter of general practice | 0.100*** | 0.032** | 0.003 |
|  | (0.027) | (0.016) | (0.030) |
| Living within 500 meter of general practic center | -0.011 | -0.061 | -0.173 |
|  | (0.129) | (0.069) | (0.136) |
| Living within 500 meter of pharmacy | 0.151*** | 0.014 | 0.005 |
|  | (0.030) | (0.018) | (0.034) |
| Living within median distance to hospital | 0.033 | 0.055** | 0.058 |
|  | (0.043) | (0.024) | (0.060) |
| Domiciliary care | 0.023 | 0.018 | 0.611*** |
|  | (0.031) | (0.019) | (0.037) |
| Personal care | 0.078** | 0.035 | 4.129*** |
|  | (0.037) | (0.024) | (0.054) |
| Nursing | -0.052 | -0.096*** | 0.188** |
|  | (0.045) | (0.032) | (0.078) |
| Personal assistance | 0.048 | -0.002 | 7.784*** |
|  | (0.090) | (0.078) | (0.199) |
| Group assistance | 0.150* | 0.048 | 15.607*** |
|  | (0.083) | (0.057) | (0.179) |
|  |  |  |  |
| Observations | 2,466,684 | 2,466,684 | 2,466,684 |
| R-squared | 0.484 | 0.545 | 0.073 |
| Neighborhood fixed effects | YES | YES | YES |

The coefficients of column 13 are multiplied by 100 and hence express percentage points.

**Appendix C: Regression results of the mechanisms**

|  | 1 | 2 | 3 |
| --- | --- | --- | --- |
|  | Use of nursing home  care on physical ground | Use of nursing home care on cognitive ground | Use of home care |
| 0/3 mix | 0.009 | 0.054 | 0.410** |
|  | (0.075) | (0.059) | (0.204) |
| 2 stars | 0.113 | 0.103* | -0.366* |
|  | (0.070) | (0.055) | (0.188) |
| 3 stars | -0.006 | -0.002 | 0.297 |
|  | (0.070) | (0.055) | (0.189) |
| 0/3 mix * Age 80-84 | -0.056 | -0.326*** | 0.135 |
|  | (0.138) | (0.116) | (0.361) |
| 0/3 mix * Age 85-89 | -0.555** | -0.215 | 0.689 |
|  | (0.241) | (0.195) | (0.584) |
| 0/3 mix * Age 90 plus | -1.023** | -0.358 | 1.590 |
|  | (0.495) | (0.406) | (1.149) |
| 2 stars * Age 80-84 | -0.346*** | -0.405*** | 0.193 |
|  | (0.126) | (0.106) | (0.327) |
| 2 stars * Age 85-89 | -1.189*** | -0.502*** | 1.262** |
|  | (0.223) | (0.179) | (0.532) |
| 2 stars * Age 90 plus | -2.368*** | -1.121*** | 3.005*** |
|  | (0.464) | (0.379) | (1.054) |
| 3 stars * Age 80-84 | -0.204 | -0.354*** | 0.604* |
|  | (0.127) | (0.107) | (0.332) |
| 3 stars * Age 85-89 | -0.851*** | -0.275 | 1.928*** |
|  | (0.224) | (0.180) | (0.536) |
| 3 stars * Age 90 plus | -1.585*** | -0.672* | 4.248*** |
|  | (0.462) | (0.378) | (1.056) |
| 2013 | -0.054** | 0.128*** | -0.357*** |
|  | (0.023) | (0.018) | (0.055) |
| 2014 | -0.433*** | 0.266*** | -0.554*** |
|  | (0.021) | (0.018) | (0.054) |
| Cholestorol reducer | -0.322*** | -0.206*** | -1.583*** |
|  | (0.020) | (0.017) | (0.054) |
| Diabetes | 0.161*** | 0.030 | 1.646*** |
|  | (0.032) | (0.027) | (0.086) |
| Astma | -0.308*** | -0.564*** | 0.299*** |
|  | (0.027) | (0.022) | (0.075) |
| Antidepressants | 0.199*** | 0.019 | 1.966*** |
|  | (0.042) | (0.038) | (0.108) |
| Antipsychotics | 3.337*** | 6.248*** | 7.395*** |
|  | (0.140) | (0.153) | (0.339) |
| Sleeping and tranquilizing tablets | -0.082 | -0.468*** | 1.541*** |
|  | (0.059) | (0.052) | (0.158) |
| ADHD and nootropics | -0.614* | -0.597** | 1.883** |
|  | (0.322) | (0.294) | (0.924) |
| Other medicines | -1.114*** | -0.877*** | -6.031*** |
|  | (0.049) | (0.046) | (0.124) |
| Log of total GP care costs in euros | 0.451*** | 0.368*** | 3.689*** |
|  | (0.030) | (0.029) | (0.062) |
| Log of total pharmaceutical care costs in euros | 0.215*** | 0.142*** | 1.313*** |
|  | (0.010) | (0.009) | (0.024) |
| Log of total oral care costs in euros | -0.024*** | -0.024*** | -0.095*** |
|  | (0.005) | (0.004) | (0.013) |
| Log of total hospital care costs in euros | -0.061*** | -0.098*** | 0.284*** |
|  | (0.004) | (0.004) | (0.010) |
| Log of total paramedical care costs in euros | -0.007 | -0.078*** | 0.424*** |
|  | (0.007) | (0.006) | (0.019) |
| Log of total technical aids costs in euros | 0.036*** | -0.033*** | 0.433*** |
|  | (0.003) | (0.003) | (0.009) |
| Log of total patient transport costs in euros | 0.039*** | -0.024*** | 0.495*** |
|  | (0.006) | (0.006) | (0.018) |
| Log of total health care costs outside the Netherlands in euros | -0.062*** | -0.063*** | -0.278*** |
|  | (0.009) | (0.006) | (0.027) |
| Log of total other care costs in euros | -0.019*** | -0.012*** | 0.003 |
|  | (0.005) | (0.004) | (0.013) |
| Gross income decile 2 | -0.091** | 0.002 | -0.614*** |
|  | (0.038) | (0.031) | (0.105) |
| Gross income decile 3 | -0.075* | 0.139*** | -1.148*** |
|  | (0.039) | (0.033) | (0.106) |
| Gross income decile 4 | -0.117*** | 0.117*** | -2.126*** |
|  | (0.041) | (0.034) | (0.109) |
| Gross income decile 5 | -0.159*** | 0.109*** | -2.713*** |
|  | (0.042) | (0.036) | (0.112) |
| Gross income decile 6 | -0.188*** | 0.107*** | -3.142*** |
|  | (0.044) | (0.038) | (0.117) |
| Gross income decile 7 | -0.164*** | 0.112*** | -3.384*** |
|  | (0.047) | (0.041) | (0.124) |
| Gross income decile 8 | -0.248*** | 0.116*** | -3.530*** |
|  | (0.051) | (0.045) | (0.136) |
| Gross income decile 9 | -0.180*** | 0.173*** | -3.629*** |
|  | (0.058) | (0.052) | (0.153) |
| Gross income decile 10 (highest) | -0.226*** | 0.187*** | -4.049*** |
|  | (0.066) | (0.061) | (0.174) |
| Financial wealth decile 2 | 0.086* | -0.022 | 0.565*** |
|  | (0.046) | (0.040) | (0.123) |
| Financial wealth decile 3 | 0.100** | -0.019 | 0.579*** |
|  | (0.046) | (0.040) | (0.121) |
| Financial wealth decile 4 | 0.199*** | 0.020 | 0.424*** |
|  | (0.046) | (0.039) | (0.120) |
| Financial wealth decile 5 | 0.168*** | -0.010 | 0.212* |
|  | (0.045) | (0.039) | (0.118) |
| Financial wealth decile 6 | 0.108** | 0.020 | 0.220* |
|  | (0.045) | (0.039) | (0.118) |
| Financial wealth decile 6 | 0.203*** | 0.037 | 0.250** |
|  | (0.045) | (0.039) | (0.117) |
| Financial wealth decile 8 | 0.145*** | 0.029 | 0.363*** |
|  | (0.046) | (0.040) | (0.120) |
| Financial wealth decile 9 | 0.059 | -0.041 | 0.121 |
|  | (0.046) | (0.040) | (0.119) |
| Financial wealth decile 10 (highest) | -0.105** | -0.224*** | 0.137 |
|  | (0.048) | (0.042) | (0.124) |
| Male | -0.045** | 0.032** | -1.070*** |
|  | (0.018) | (0.016) | (0.048) |
| Dutch | 0.385*** | 0.358*** | 1.326*** |
|  | (0.035) | (0.030) | (0.087) |
| Having a partner | -0.216*** | 0.291*** | -0.726*** |
|  | (0.021) | (0.018) | (0.057) |
| Having children living at home | -0.258*** | -0.236*** | 0.083 |
|  | (0.040) | (0.036) | (0.104) |
| Age 80-84 | 0.831*** | 0.854*** | 4.073*** |
|  | (0.124) | (0.104) | (0.320) |
| Age 85-89 | 2.846*** | 1.691*** | 8.656*** |
|  | (0.218) | (0.174) | (0.516) |
| Age 90plus | 6.026*** | 3.290*** | 13.380*** |
|  | (0.449) | (0.366) | (1.008) |
| Having children | -0.193*** | -0.016 | -0.487*** |
|  | (0.030) | (0.026) | (0.074) |
| Home owner | -0.100*** | -0.081*** | -1.297*** |
|  | (0.021) | (0.018) | (0.056) |
| Living within 500 meter of supermarket | 0.041* | -0.010 | 0.083 |
|  | (0.024) | (0.020) | (0.061) |
| Within 500 meter of general practice | 0.023 | -0.010 | 0.222*** |
|  | (0.026) | (0.023) | (0.067) |
| Living within 500 meter of general practic center | -0.155 | -0.115 | -0.039 |
|  | (0.117) | (0.101) | (0.307) |
| Living within 500 meter of pharmacy | -0.008 | 0.049** | -0.021 |
|  | (0.029) | (0.025) | (0.074) |
| Living within median distance to hospital | 0.091* | -0.008 | -0.051 |
|  | (0.052) | (0.045) | (0.133) |
| Domiciliary care | 0.647*** | 0.129*** |  |
|  | (0.032) | (0.027) |  |
| Personal care | 3.040*** | 3.030*** |  |
|  | (0.047) | (0.042) |  |
| Nursing | 0.588*** | 0.035 |  |
|  | (0.070) | (0.063) |  |
| Personal assistance | 6.839*** | 9.804*** |  |
|  | (0.184) | (0.188) |  |
| Group assistance | 11.110*** | 15.719*** |  |
|  | (0.166) | (0.170) |  |
| Constant | -1.732*** | -1.721*** | -13.378*** |
|  | (0.175) | (0.160) | (0.382) |
|  |  |  |  |
| Observations | 2,567,430 | 2,553,844 | 1,789,382 |
| R-squared | 0.061 | 0.099 | 0.056 |
| Neighborhood fixed effects | YES | YES | YES |
| Specification | OLS | OLS | OLS |

All specifications include health controls, personal characteristics, and neighborhood characteristics. The coefficients are multiplied by 100 and hence express percentage points. ***p<0.01, **p<0.05, *p<0.1.
